# Editorial: The Biochemistry of Amyloids in Neurodegenerative Diseases, Volume I

**DOI:** 10.3389/fnins.2021.819481

**Published:** 2021-12-16

**Authors:** Cláudio M. Gomes, Wolfgang Hoyer, Jinghui Luo

**Affiliations:** ^1^Faculdade de Ciências, Biosystems and Integrative Sciences Institute, Universidade de Lisboa, Lisbon, Portugal; ^2^Departamento de Química e Bioquímica, Faculdade de Ciências, Universidade de Lisboa, Lisbon, Portugal; ^3^Institute of Physical Biology, Heinrich Heine University Düsseldorf, Düsseldorf, Germany; ^4^Institute of Biological Information Processing (IBI-7) and Jülich Center for Structural Biology, Forschungszentrum Jülich, Jülich, Germany; ^5^Department of Biology and Chemistry, Paul Scherrer Institute, Villigen, Switzerland

**Keywords:** protein aggregation, tau, synuclein, SOD1, Alzheimer's disease, Parkinson's disease, aggregation inhibitors, amyloid beta

Protein aggregation and formation of amyloids is a key pathological hallmark across multiple neurodegenerative diseases such as Alzheimer's disease (AD) and Parkinson's Disease (PD). Understanding the structural conversions underlying the formation of amyloid oligomers and fibrils, as well as the relevant molecular mechanisms that dictate the rate and type of aggregation, is critical not only to establish the underlying fundamental biological processes related to amyloid aggregation *in vivo*, but also to envision the design of mechanism-based and/or structure-oriented therapeutics against amyloid-related neurodegenerative diseases. This Research Topic and e-book comprises a series of original papers and updated reviews that present advances in this direction and highlight trends in the field, covering aspects related to mechanisms of amyloid formation and toxicity, the role of post translational modifications, and inhibitors of protein aggregation.

The formation of fibrillar aggregates is triggered by aggregation prone regions within polypeptides whose misfolding will trigger self-assembly into an ordered amyloid core, now elucidated in several atomic-resolution structures of the amyloid fold. However, as reviewed by Ulamec et al. the regions that flank such amyloid cores, which often form dynamic fuzzy coats around the amyloid core, play key roles as modulators of function, toxicity, and aggregation. In this comprehensive analysis, the authors present and discuss the flanking regions that either protect or accelerate aggregation and provide a conceptual framework to understand their roles in regulating the mechanism of self-assembly, interactions with other amyloidogenic proteins, membranes, chaperones, and RNA. The authors convincingly argue that investigations on these flanking regions will lead to a better understanding of the molecular mechanism of fibril formation and to the identification of new targets for drug development beyond the ordered amyloid core and the aggregation hotspots.

Superoxide dismutase 1 (SOD1) typifies a protein whose destabilization triggers amyloid fibrillation as in amyotrophic lateral sclerosis. Destabilization of the SOD1 homodimer due to loss of the catalytic Cu and Zn ions greatly destabilizes the protein, favoring reduction of a conserved intra-molecular disulfide that aggravates dimer dissociation, resulting in exposure of otherwise solvent shielded aggregation prone regions. However, SOD1 contains two additional distal cysteines whose role in the aggregation pathway remained unclear. Koo et al. here report an investigation on the role of free thiols and disulfide exchange in the aggregation of SOD1, concluding that the reduction status modulates the formation of a novel SOD1 oligomer with mixed disulfides, with implications in fibril formation. Amyloid induced toxicity is known to be also caused by amyloid pores. Venko et al. present predictive approaches to assess the propensity of amyloid forming proteins to form transmembrane channels, suggesting that many have such potential and that oligomerization and conformational transitions in lipid rafts may be critical common events.

Post translational modifications are known to impact in the formation of protein aggregates and to influence their structure and toxicity. However, the repertoire of disease-associated modifications is vast, and its study presents several challenges. Two contributions in this Research Topic are focused on post translational modifications of proteins implicated in neurodegeneration. Kumar et al. report the development of novel phosphorylation-state specific antibodies that can differentiate Aβ peptides depending on the phosphorylation state of Ser26. Such site- and phosphorylation state-specific Aβ antibodies have the potential to be employed as tools in studies aimed at investigating the spatio-temporal deposition of different Aβ variants in transgenic mouse models and human AD brains. Kametani et al. have compared common and disease-specific post-translational modifications of pathological tau associated with multiple tauopathies. For this, detergent insoluble tau inclusions prepared from brains of patients suffering from a wide range of tauopathies were investigated, resulting in the identification of new tau modifications and in the hypothesis that differences in PTMs may be related to the observed differences in the structures of tau filament core structures.

The ability to modulate amyloid formation by selectively targeting aggregation-prone proteins implicated in neurodegenerative diseases is of great interest in drug discovery programmes. Protein-based biologics are of particular relevance, as antibodies or peptides have the potential to be engineered toward increased molecular recognition and specificity. Reflecting this emerging trend, several contributions in this Research Topic are focused on the use of peptides to inhibit protein aggregation and toxicity. Along these lines, Mamsa and Meloniv review the potential of cationic arginine-rich peptides (CARPS) as an emergent class of promising neurotherapeutics in AD capable to prevent toxic aggregation of Aβ and tau. Departing from an analysis of the structural and physicochemical characteristics and of the emerging properties of the arginine moiety, the authors present a thorough overview of the types and mechanisms of action of several inhibitory peptides that attenuate the aggregation and cytotoxicity of Aβ and tau and may even prove to be useful to decrease soluble cytotoxic oligomers. Also in the context of AD, Ikenoue et al. describe the rational design of bicyclic peptides that target various epitopes along the most amyloidogenic region of the Aβ sequence. By combining kinetic and structural approaches, the authors show that these compounds have the potential to remodel the aggregation of Aβ by redirecting it toward non-fibrillar species. Indeed, at sub-stoichiometric levels the bicyclic peptides delay the condensation of Aβ and the subsequent formation of fibrils, a process which is inhibited at high compound concentrations. Torpey et al. undertake a study focused on the inhibition of α-synuclein aggregation by the 4554W peptide, whose mechanism of action is investigated from a structural perspective. The authors use NMR to probe the interaction and conclude that 4,554W associates with a partially aggregated form of α-synuclein, with enhanced association occurring over time. Also, they report that this peptide reduces the formation of α-synuclein fibrils resulting from PD-associated mutations and that it disaggregates pre-formed fibrils, as inferred from an effect over fibril length. The article by Popova et al. describes a yeast based high-throughput screening for short peptides that inhibit α-synuclein aggregation, leading to the identification of several peptides capable to supress aggregation and toxicity in living cells. Subsequent *in vitro* assays using two of these peptides established that they significantly inhibit α-synuclein oligomerization and aggregation at sub-stoichiometric molar ratios and that some are effective in inhibiting the formation of early oligomers. Finally, Pagano et al. reviewed natural compounds shown to interfere with Aβ aggregation by direct interaction with Aβ peptide and whose inhibitory mechanism has been investigated by means of biophysical and structural biology experimental approaches. This can provide a rationale for the selection of natural compounds as molecular scaffolds for the design of new therapeutic strategies against the progression of early and late stages of AD.

## Author Contributions

All authors have contributed to writing of the editorial and all have handled submissions to the Research Topic as topic editors.

## Conflict of Interest

The authors declare that the research was conducted in the absence of any commercial or financial relationships that could be construed as a potential conflict of interest.

## Publisher's Note

All claims expressed in this article are solely those of the authors and do not necessarily represent those of their affiliated organizations, or those of the publisher, the editors and the reviewers. Any product that may be evaluated in this article, or claim that may be made by its manufacturer, is not guaranteed or endorsed by the publisher.

